# Telemedicine networks for acute stroke: An analysis of global coverage, gaps, and opportunities

**DOI:** 10.1177/17474930241298450

**Published:** 2024-11-15

**Authors:** Christine Tunkl, Ayush Agarwal, Emily Ramage, Faddi Saleh Velez, Tamer Roushdy, Teresa Ullberg, Linxin Li, Leonardo A Carbonera, Abdul Hanif Khan Yusof Khan, Bogdan Ciopleias, Zhe Kang Law, Aristeidis H Katsanos, Mirjam R Heldner, Maria Khan, Sarah Matuja, Matias J Alet, Javier Lagos-Servellón, Jatinder S Minhas, Susanna M Zuurbier, Maria Giulia Mosconi, Radhika Lotlikar, Ahmed Elkady, Stefan T Gerner, Shirsho Shreyan, Alexandra Krauss, Christoph Gumbinger, Padma Srivastava, Pawel Kiper, Robin Ohannessian, Anne Berberich, Gisele Sampaio Silva, Anna Ranta

**Affiliations:** 1 Department of Neurology, University Hospital Heidelberg, Heidelberg, Germany; 2 Department of Neurology, All India Institute of Medical Sciences, New Delhi, New Delhi, India; 3 The Florey Institute of Neuroscience and Mental Health, and Western Health, Parkville, VIC, Australia; 4 Department of Neurology, The University of Oklahoma Health Sciences Center, Oklahoma City, OK, USA; 5 Department of Neurology, Faculty of Medicine, Ain Shams University, Cairo, Egypt; 6 Department of Neurology, Skane University Hospital, Clinical Sciences, Lund University, Lund, Sweden; 7 Wolfson Centre for Prevention of Stroke and Dementia, Nuffield Department of Clinical Neurosciences, University of Oxford, Oxford, UK; 8 Department of Neurology and Neurosurgery, Hospital Moinhos de Vento, Porto Alegre, Brazil; 9 Department of Neurology, Faculty of Medicine and Health Sciences, Universiti Putra Malaysia, Serdang, Malaysia; 10 Department of Neurology, Faculty of Medicine, Transilvania University of Brasov, Brasov, Romania; 11 Department of Medicine, Faculty of Medicine, National University of Malaysia (UKM), Kuala Lumpur, Malaysia; 12 Department of Medicine (Neurology), McMaster University and Population Health Research Institute, Hamilton, ON, Canada; 13 Department of Neurology, Inselspital, University Hospital and University of Bern, Bern, Switzerland; 14 Department of Neurology, Rashid Hospital, Mohammed Bin Rashid University of Medical and Health Sciences, Dubai, UAE; 15 Department of Medicine, Weill Bugando School of Medicine, Mwanza, Tanzania; 16 Department of Neurology, Comprehensive Stroke Center, Fleni, Ciudad de Buenos Aires, Argentina; 17 Hospital CEMESA/HNMCR, San Pedro Sula, Honduras; 18 Cerebral Haemodynamics in Ageing and Stroke Medicine (CHiASM), Department of Cardiovascular Sciences, University of Leicester, Leicester, UK; 19 NIHR Leicester Biomedical Research Centre, BHF Cardiovascular Research Centre, Glenfield Hospital, Leicester, UK; 20 Department of Neurology, University Hospital Antwerp, Edegem, Belgium; 21 Internal Vascular and Emergency Medicine—Stroke Unit, University of Perugia, Perugia, Italy; 22 Department of Neurology, Deenanath Mangeshkar Hospital and Research Center, Pune, India; 23 Saudi German Hospitals, Jeddah, Saudi Arabia; 24 Department of Neurology, University Hospital Erlangen, Erlangen, Germany; 25 Rajshahi Medical College, Rajshahi, Bangladesh; 26 Healthcare Innovation Technology Lab, San Camillo IRCCS, Venice, Italy; 27 Vickino Institute of Telehealth (VIT), Vickino, Yerevan, Armenia; 28 Department of Neurology, Klinikum Ludwigshafen, Ludwigshafen, Germany; 29 Albert Einstein Hospital, Sao Paulo, Brazil; 30 Departamento de Neurologia da Universidade Federal de São Paulo (UNIFESP), São Paulo, Brazil; 31 Department of Medicine, University of Otago, Wellington, New Zealand

**Keywords:** Telestroke, networks, guidelines, stroke, healthcare implementation

## Abstract

**Background:**

Despite the proven efficacy of telestroke in improving clinical outcomes by providing access to specialized expertise and allowing rapid expert hyperacute stroke management and decision-making, detailed operational evidence is scarce, especially for less developed or lower income regions.

**Aim:**

We aimed to map the global telestroke landscape and characterize existing networks.

**Methods:**

We employed a four-tiered approach to comprehensively identify telestroke networks, primarily involving engagement with national stroke experts, stroke societies, and international stroke authorities. A carefully designed questionnaire was then distributed to the leaders of all identified networks to assess these networks’ structures, processes, and outcomes.

**Results:**

We identified 254 telestroke networks distributed across 67 countries. High-income countries (HICs) concentrated 175 (69%) of the networks. No evidence of telestroke services was found in 58 (30%) countries. From the identified networks, 88 (34%) completed the survey, being 61 (71%) located in HICs. Network setup was highly heterogeneous, ranging from 17 (22%) networks with more than 20 affiliated hospitals, providing thousands of annual consultations using purpose-built highly specialized technology, to 11 (13%) networks with fewer than 120 consultations annually using generic videoconferencing equipment. Real-time video and image transfer was employed in 64 (75%) networks, while 62 (74%) conducting quality monitoring. Most networks established in the past 3 years were located in low- and middle-income countries (LMICs).

**Conclusion:**

This comprehensive global survey of telestroke networks found significant variation in network coverage, setup, and technology use. Most services are in HICs, and a few services are in LMICs, although an emerging trend of new networks in these regions marks a pivotal moment in global telestroke care. The wide variation in quality monitoring practices across networks, with many failing to report key performance metrics, underscores the urgent need for standardized, resource-appropriate, quality assurance measures that can be adapted to diverse settings.

## Introduction

Telemedicine to provide remote expert stroke care has demonstrated significant effectiveness in improving clinical outcomes through the rapid administration of thrombolytic therapy and timely decision-making in remote areas that lack vascular neurology expertise, both of which are critical for effective stroke management.^1–3^ Despite robust evidence of efficacy and safety, detailed knowledge of the operational mechanisms of telestroke networks remains scarce.^4^ The research findings regarding telestroke networks rely predominantly on evaluating networks in high-income countries (HICs). There is no comprehensive overview of telestroke networks worldwide.^4,5^ Furthermore, extensive data on telestroke networks in the United States and Germany^6–8^ contrast sharply with the limited information from other parts of the world, suggesting that telestroke evidence should not be readily generalized across contexts^9^ and highlighting the knowledge gap for regions that have yet to establish telestroke systems.

Current guidelines and recommendations provide clear guidance on the framework and requirements for telestroke networks. However, these guidelines are primarily based on research, expert opinions, and experiences from HICs and may not be universally applicable.^10–13^ As a result, low- and middle-income countries (LMICs) are left without practical, context-specific guidance on initiating and monitoring high-quality telestroke care.

### Aim

Our project aimed to map the global telestroke landscape and elucidate current practices to provide a database for the development of universally applicable recommendations to facilitate telestroke implementation across the globe.

## Method

### Definition of telestroke care

For our study, we defined telestroke networks as those designed for use by healthcare professionals between hospitals operating within the emergency and acute inpatient hospital setting (<3 days after onset) and are based on any formal processes recognized by the hospitals.

We excluded telerehabilitation networks and direct physician-to-patient telemedicine consultations, private consultations, guidance, mentorship, irregular or friendship-based advice, or communication between residents and consultants within a hospital.

We defined a telestroke system of care as the provision of stroke expertise to remote sites with limited or no vascular neurology coverage, allowing rapid evaluation and treatment decisions by skilled and experienced stroke physicians through either video examination or voice-only approaches.

### Network identification

The identification of telestroke networks involved a four-step approach. Step 1: A top-down approach, leveraging the personal connections with scientific or clinical leaders of the World Stroke Organization Future Leaders research team, which consists of participants from 34 countries evenly distributed across global regions. In addition, the first step included contacting Angels’ Initiative country coordinators to confirm the availability of telestroke networks in their respective countries.^14^

Step 2: A short pre-survey was distributed among members of regional and global stroke societies, asking participants to note any available networks in their region.

Step 3: In countries where no direct contacts and no responses via the pre-survey were available, extensive Google and PubMed searches were conducted using the terms “stroke” or “neurology” along with the country name to identify any scientific initiatives or researchers in each country. At least two attempts were made to contact representatives and organizations in each country.

Step 4: The list of networks was reviewed for comprehensiveness by international stroke experts.

### Survey design

Seven international stroke researchers from five different world regions initially developed a draft questionnaire. A pilot was conducted in which the co-authors conducted a test run of the survey and provided additional feedback for the final release of the survey. The final questionnaire consisted of 39 questions with an estimated completion time of 15 minutes. The survey was divided into four sections: structure, process, outcome indicators, and self-perceived strengths and limitations (see Supplement 1 for the questionnaire).

Study data were collected and managed using the Research Electronic Database Capture System (REDCap), a secure, web-based application hosted at the All India Institute of Medical Sciences (AIIMS).^15^

### Survey distribution

The first wave of the survey was distributed on 29 January 2024 to the coordinators of all identified telestroke networks. The second wave was distributed on 2 May 2024 to the coordinators of additionally identified telestroke networks. Participants provided informed consent by completing the survey, which included a clear statement at the beginning explaining the study’s purpose and voluntary nature. Local research ethics approval was obtained through the Ethics Committee, University Hospital of Heidelberg (S-473/2023).

### Statistical analysis

Variables are presented as frequencies and percentages. Continuous variables are presented as medians and interquartile ranges.

The classification of countries is derived from two sources: The Institute for Health Metrics and Evaluation (IHME) Global Burden of Disease (GBD) study categorizes seven super-regions based on epidemiological similarity and geographical proximity,^16^ which are High-Income, Latin America and the Caribbean, sub-Saharan Africa, North Africa and Middle East, South Asia, Southeast Asia and East Asia and Oceania, Central Europe and Eastern Europe, and Central Asia.

The World Bank classifies countries by income level into high-income (HIC), upper-middle-income (UMIC), lower-middle-income (LMIC), and low-income (LIC) categories and uses the Gross National Income (GNI) per capita as the primary metric.^17^ Data analysis was performed using SPSS version 29.0.

## Results

### Network identification

**Figure 1.**
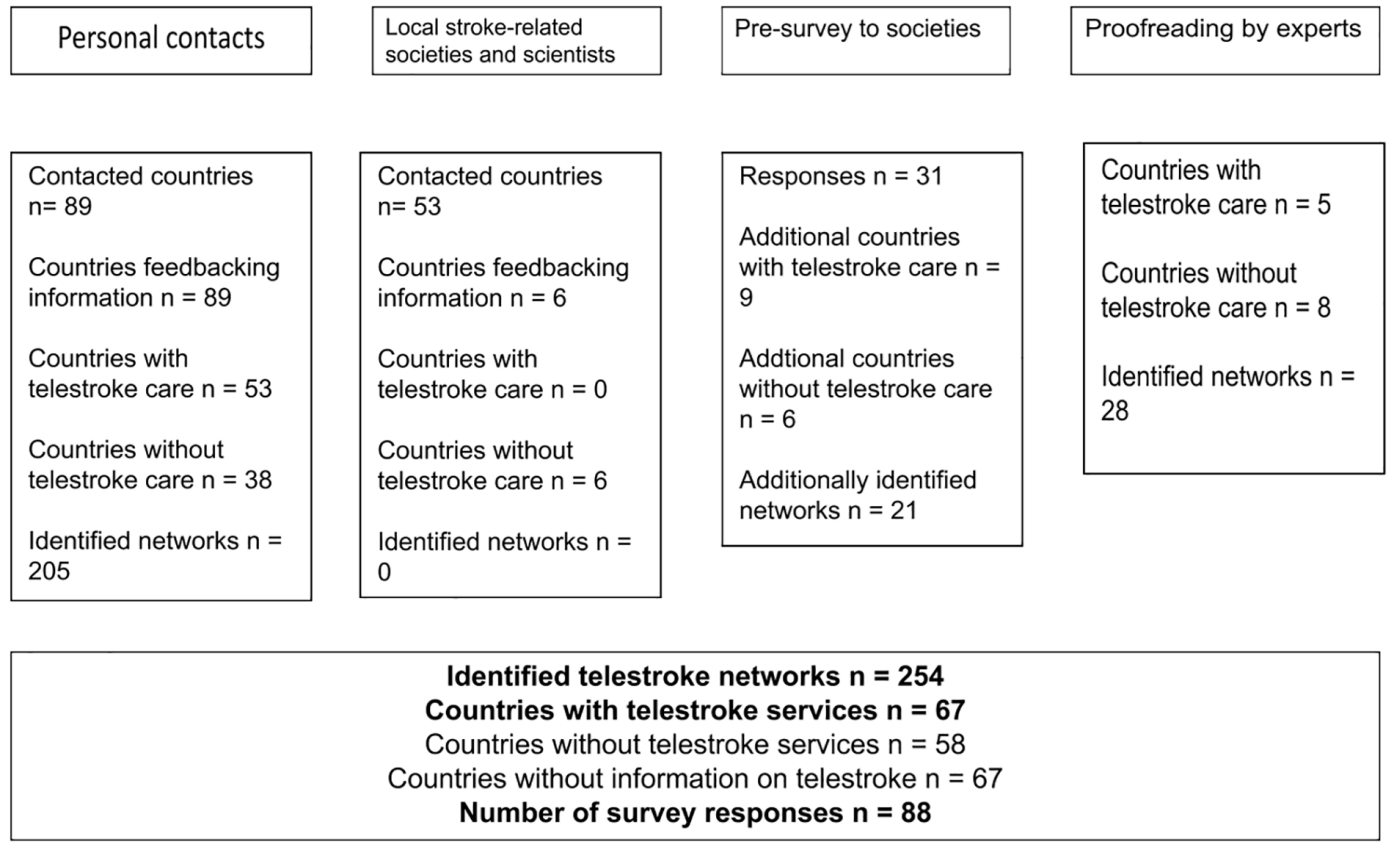
Telestroke network identification process.

In total, 254 telestroke networks from 67 countries were identified and 58 countries reported the absence of telestroke care to the best of their knowledge. Most networks were identified by using personal contacts on the country level (*n* = 205 from 53 countries) and a brief survey of members of stroke societies (*n* = 21 from 9 countries). Proof-reading by experts revealed additional 28 telestroke networks from five countries (see Figure 1 and Supplementary File 2).

### Global coverage of telestroke care

**Figure 2.**
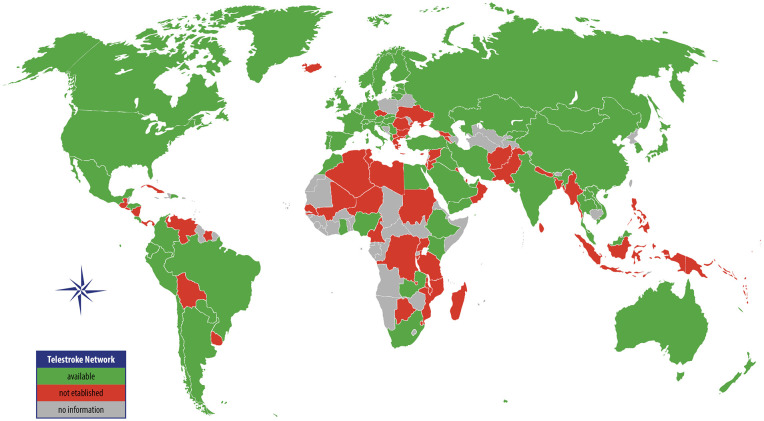
Global coverage of acute telestroke networks.

The global distribution of 254 telestroke networks spans 67 countries, with the majority concentrated in HICs, accounting for 188 networks (74.1%) (see Figure 2 and Supplementary File 3). UMICs follow with 46 networks (18.1%). In LMICs, 19 networks (7.5%) were identified, and a single network (0.3%) was reported in a low-income country. Conversely, the countries reporting the absence of telestroke services were predominantly LMICs (*n* = 21; 36.2%) and UMICs (*n* = 14; 24.1%).

According to the classification used in the GBD study by IHME, which groups countries into seven super-regions based on epidemiological similarity and geographical proximity,^16^ telestroke coverage is led by HICs with 175 networks (68.6%), followed by Central Europe, Eastern Europe, and Central Asia (EURCA) with 27 networks (10.6%), and Latin America and the Caribbean (LATAM) with 16 networks (6.3%). The countries with the highest number of identified telestroke networks are the United States (*n* = 55; 21.7% of all networks), Germany (*n* = 19; 7.5%) and Spain (*n* = 13; 5.1%).

### Survey

**Figure 3.**
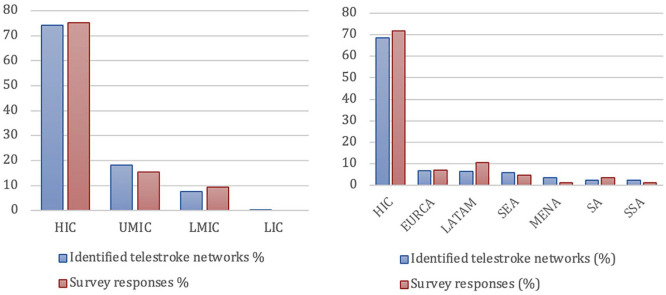
Comparison of identified telestroke networks and survey responses in percentage by (a) World Bank country classification by income level and (b) GBD super-region. Number of networks in percentage. GBD: Global Burden of Disease; HIC: high-income countries; UMIC: upper-middle income countries; LMIC: lower-middle income countries; LIC: low-income countries; EURCA: Central Europe, Eastern Europe and Central Asia; LatAm: Latin America and the Caribbean; SEARO: South-East-Asia and Oceania; MENA: Middle-East and North-Africa; SA: South-Asia; SSA: sub-Saharan Africa.

The survey was completed by 88 telestroke networks from 31 countries, giving a response rate of 34% from all invited network coordinators. The response rate of the survey mirrors the global distribution of telestroke networks (see Figure 3). Two questionnaires had to be excluded: one was a duplicate and another focused exclusively on rehabilitation.

The main respondents were network coordinators (41.9%) or telestroke providers (38.4%). Most respondents had been involved in their network for over five years (59.3%), and less than 6% had under one year of experience with telestroke.

### Structural characteristics of telestroke networks

The median duration of operation of all telestroke networks was 9 years (5–12) at the time of survey response, with the median duration of operation of HIC telestroke networks 7 years greater than lower-and middle-income regions (10 (7–14) years versus 3 (2–5) years, respectively). Of the networks established in the past three years, 14 (87.5%) are in non-high-income regions. Conversely, 34 (92%) networks established more than ten years ago were in HICs (see Table 1).

**Table 1.** Structural characteristics of networks by GBD super-region.

	Total *n* (%)	HIC	EURCA	LATAM	SEARO	MENA	SA	SSA
	85 (100%)	61 (71.8%)	6 (7.1%)	9 (10.6%)	4 (4.7%)	1 (1.2%)	3 (3.5%)	1 (1.2%)
Years since network establishment (median (IQR))	9 (5–12)	10 (7–14)	5	3.5	2.5	2	2.3	n.a.
Years existing								
⩽3 years	16	2	2	6	2	1	3	0
4–9 years	22	19	2	1	0	0	0	0
⩾10 years	37	34	2	1	0	0	0	0
Organizational model								
Hub & spoke	56 (72.1%)	45	5	5	4	0	1	n.a.
Hubless/spoke-to-spoke	14 (18.6%)	11	0	4	0	0	1	n.a.
Other		5	1	0	0	1	1	n.a.
No. of hospitals involved in a network	9 (5–16.5)	10 (6–18)	5 (3.5–13)	4 (3–5)	8.5 (5.5–17.5)	46	40	5
No of hospitals in hub & spoke networks	9.5 (5–14.5)	10 (6–14)	5 (3.5–13)	4.5 (4–24)	8.5 (5.5–17.5)	46	40	5
No of hospitals in hubless networks	7.5 (3–32)	9 (7–45)	n.a.	3 (2–3)	n.a.	n.a.	n.a.	n.a.
No of consultations/month/network								
<10 consultations/month	11 (12.9%)	5	2	3	0	0	1	0
10–50 consultations/month	21 (24.7%)	11	3	5	1	0	1	0
50–100 consultations/month	14 (16.5%)	12	1	0	1	0	0	0
>100 consultations/month	32 (37.6%)	29	0	0	1	1	1	0
Operating hours								
Out-of-working hours	6 (7%)	2	0	1	0	0	1	1
24/7	79 (91.1%)	58	6	8	4	1	2	0
Setting of providing hospital								
Academic, comprehensive center	63 (74.4%)	45	5	8	2	0	3	0
Non-academic comprehensive center	10 (11.8%)	6	1	1	2	0	0	0
Secondary care center	5 (5.9%)	4	0	0	0	0	0	1
No originating hospital exists	3 (3.5%)	3	0	0	0	0	0	0
Setting of remote/spoke hospital								
Secondary care center	54 (63.5%)	39	3	5	4	1	2	0
Primary care center	37 (43.5%)	27	2	4	1	0	2	1
Non-academic comprehensive center	11 (12.9%)	8	1	1	1	0	0	0
Academic, comprehensive center	7 (8.2%)	6	1	0	0	0	0	0

Number of networks in absolute numbers.

HIC: High-income regions; EURCA: Central Europe, Eastern Europe and Central Asia; LatAm: Latin America and the Caribbean; SEARO: South-East-Asia and Oceania; MENA: Middle East and North-Africa; SA: South-Asia; SSA: sub-Saharan Africa.

Fifty-six (72%) of responding networks use a “Hub & Spoke” organization model, where stroke specialists at a central hub advise regional spokes before local treatment or transfer to the hub. Fourteen (19%) used a hubless model, where teleconsultations are performed between spoke hospitals without one central hospital.

The median number of hospitals per network is 9 (5−17) ranging from one affiliated to 70 affiliated hospitals. Networks having more than 50 affiliated hospitals are found in the United States, Australia, Spain, Canada, and India. “Hub & Spoke” networks tend to have slightly more hospitals per network than hubless networks (*n* = 10 (5–15) versus *n* = 8 (3–32) affiliated hospitals, see Table 1).

Forty-six (54%) telestroke networks report conducting more than 50 consultations per month, with 32 (37.6%) of those conducting more than 100 (*n* = 29, 90.1% in HICs as indicated in Table 1). Seventy-nine (91.1%) telestroke networks operate in a 24/7 mode and six networks (7%) offer telemedical support out-of-working hours only. Sixty-four (75%) networks operate with a part-time or supernumerary working model. In comparison, 13% of telestroke physicians work as telestroke clinicians as a full-time job, and these are mainly in networks with more than 100 consultations per month. Seventy (82%) telestroke networks employ consultant neurologists with more than 5 years of clinical experience as telestroke physicians. Fifty-nine (69%) telestroke services receive consultation requests primarily from emergency physicians, while 47 (55%) of services reported requesters primarily from resident doctors. In 63 (74%) networks, hospitals providing telestroke consultations were academic comprehensive centers, 10 (12%) networks were guided by non-academic comprehensive centers, and 5 (6%) by non-comprehensive care centers.

Fifty-four (63.5%) of the remote or spoke hospitals were secondary care centers and 37 (43.5%) networks include primary care centers in their telestroke networks.

### Differences between network models by network size

Regarding the telestroke network size, 35 (46%) networks have 6–20 affiliated hospitals, 24 (32%) have 0–5 affiliated hospitals, and 17 (22%) have more than 20 affiliated hospitals, as were categorized as medium-sized, small-sized, and large-sized, respectively. Small-sized networks are reported from different GBD super-regions (HIC *n* = 13 (54%), LATAM *n* = 6 (25%), EURCA *n* = 3 (13%)). Medium-sized and large-sized networks are mainly found in HICs (*n* = 31 (89% and *n* = 13 (77%), respectively (Table 2)). Large-sized networks have a standard operational procedure (SOP) available more often compared with small-sized networks (*n* = 16 (94%) versus *n* = 18 (75%)) and have a higher prevalence of performing video examination (*n* = 17 (100%) versus *n* = 18 (76%)).

**Table 2.** Characteristics of networks by network size.

	*n* = 76	Small (⩽5 affiliated hospitals)	Medium (6–20 hospitals)	large ⩾ 20 affiliated hospitals
		*n* = 24 (32%)	*n* = 35 (46%)	*n* = 17 (22.4%)
Average number of hospitals in the network (median, IQR)		3 (2–5)	10 (8–12)	30 (21–46)
GBD super-region	HIC	13 (54.2%)	31 (88.6%)	13 (76.5%)
	LATAM	6 (25%)	0 (0%)	1(5.9%)
	EURCA	3 (12.5%)	2 (5.7%)	0 (0%)
	SEARO	1 (4.2%)	2 (5.7%)	1(5.9%)
	Other	1 (4.2%)	0 (0%)	2 (11.8%)
Years since the network was established		5 (2–8)	10 (7–13)	10 (5–18)
No of consultations/month/network	<10 consultations/month	8 (33.3%)	1 (2.9%)	0 (0%)
	10–50 consultations/month	10 (41.7%)	8 (22.9%)	0 (0%)
	50–100 consultations/month	2 (8.3%)	9 (25.7%)	2 (11.8%)
	>100 consultations/month	1 (4.2%)	14 (40%)	14 (82.4%)
SOP available?	Yes	18 (75%)	27 (77.1%)	16 (94.1%)
Research activities?	Yes	12 (50%)	21 (60%)	12 (70.6%)
Quality monitoring in place?	Yes	18 (75%)	25 (71.4%)	13 (76.5%)
Components	Video examination	18 (76%)	25 (71.4%)	17 (100%)
	Video examination and imaging transfer	16 (66.7%)	26 (74.3%)	17 (100%)

GBD: Global Burden of Disease study; HIC: high-income regions; EURCA: Central Europe, Eastern Europe and Central Asia; LatAm: Latin America and the Caribbean; SEARO: South-East-Asia and Oceania, based on Global Burden of Disease super-regions. SOP: Standardized operational procedure.

### Purpose of telestroke networks

The main indicated reasons for telestroke consultations are emergency consultations for hyperacute stroke cases (*n* = 74; 86% of all networks), thrombolysis (*n* = 76; 88%), thrombectomy (*n* = 62; 72%), and/or transfer to thrombectomy centers (*n* = 70; 81%). Telestroke networks are also frequently used to discuss difficult cases not requiring hyperacute reperfusion therapy (*n* = 59; 69%), to consult on neurosurgical cases (*n* = 56; 65%), including transfer to neurosurgical interventions (*n* = 57; 66%), or for transfer to a stroke unit or neurointensive care unit (*n* = 51; 59%). Stroke unit monitoring and follow-up are provided by 24 telestroke networks (28%). Research activities are undertaken in 51 (60%) of all networks (63% of networks in HIC vs 50% of all networks in the other regions).

### Components of a telestroke consultation

Fifty-one (59%) networks state that telestroke consultations last between 5 and 20 minutes, with two (2%) networks requiring more than 45 minutes.

**Figure 4.**
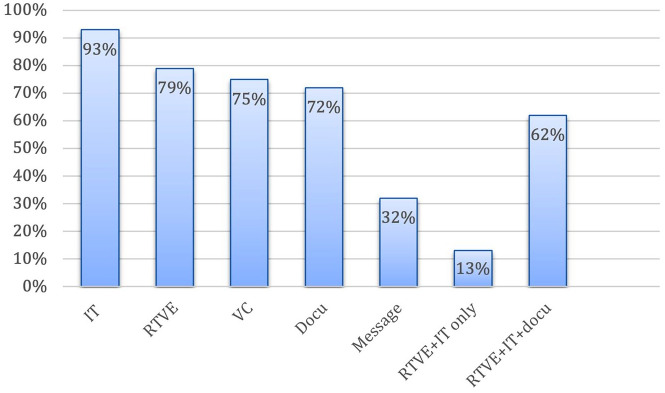
Components of a telestroke consultation. IT: imaging transfer; RTVE: real-time-video examination; VC: voice call; Docu: written documentation; Message: text messaging.

Nearly all networks include imaging transfer (*n* = 79, 93%), but 18 (21%) networks from various regions perform consultations without video examination (Figure 4). Fifty-three (62%) networks have video examination, image transfer, and written documentation as typical components of a consultation.

While image transfer is mostly done through the regular Picture Archiving and Communication System (PACS) (*n* = 61, 72%), 22 networks use a dedicated image transfer application (26%) or dedicated image transfer systems (*n* = 14, 17%).

Video examination is performed by dedicated software applications for telestroke care (*n* = 39, 46%), by specialized hardware camera devices (*n* = 29; 34%), or by commercial, non-medical messaging applications (e.g. WhatsApp^®^) (*n* = 17; 20%). Examples cited for video examination are dedicated computers on wheels with Zoom software, Teladoc Health’s RP Vita robot, or a Vidyoconnect application.

In 38 (45%) of the surveyed networks, documentation is done through the regular hospital system and in 26 (31%) through a specialized stand-alone telestroke application. Other options described for documentation include REDCap^®^ forms, Microsoft^®^ Shared Folder, Microsoft Forms^®^ application, template within email, or Epic^®^ Telestroke Navigator.

Commercial, non-medical messaging applications are used for image transfer in 9 (11%) telestroke networks and for video examinations in 17 (20%) networks. Notably, of all networks that utilize non-medical applications for video examinations, 10 (59%) are in non-HICs. HICs use more high-cost specialized technologies, while other regions rely more on messaging applications.

### Challenges and user satisfaction

The survey identified several common challenges faced by telestroke networks. Technical difficulties related to image transfer and communication were reported by 37 (43%) and 35 (41%) networks, respectively; 34 (40%) networks cited financial constraints as a significant barrier. In addition, 24 (28%) networks identified inadequate Internet connectivity as a barrier to effective, uninterrupted telestroke care delivery. Time constraints were cited as a concern by 18 (21%) networks.

Despite these challenges, the data indicate high level of perceived effectiveness and satisfaction among network coordinators and clinical providers. An overwhelming majority (*n* = 79, 92%) of respondents cited improved patient care as a key strength of their network. Furthermore, nearly 98% of respondents reported being either satisfied or very satisfied with their network’s performance.

### Quality monitoring in telestroke

Overall, 62 (74%) networks reported some form of quality monitoring with slight variation by region or income level (47 (77%) networks in HICs and 15 (63%) networks in other GBD super-regions). Fifty-nine (69%) networks report their door-to-treatment time and 35 (41%) the recanalization rate. Among networks that did monitor door-to-needle-time (DNT; *n* = 49; 57%), the reported median was 48 (40–60) min, with a wide range of 18–98 min. This variability suggests significant differences in quality performance across networks.

Additional important outcome measures are inconsistently recorded. In-hospital mortality data and discharge National Institutes of Health Stroke Scale (NIHSS) scores are monitored by 48 (57%) and 40 (47%) networks, respectively.

## Discussion

Our study provides a global overview and characterization of telestroke networks based on responses to a worldwide survey. These data offer important insights into global telestroke network organization and performance. A total of 254 telestroke networks were identified across 67 countries, with 69% located in HICs. Coverage in non-high-income regions was markedly limited, with only 21 networks identified across South Asia, the Middle East and North Africa, and sub-Saharan Africa combined. Notably, 78% of countries reporting no telestroke networks were classified as upper-middle, lower-middle, or low-income. The lack of telestroke networks in sub-Saharan Africa was particularly striking, confirming the previous findings of stroke care challenges in this region.^18^ This study underscores significant disparities in global coverage of telestroke care, consistent with findings from the GBD study^19^ and a recent World Health Organization survey^20^ indicating inadequate access to stroke care in LMICs.

### Characteristics of telestroke networks

The survey revealed considerable heterogeneity among telestroke networks in size, operational capacity, and quality monitoring practices, with most of the available data coming from high-income regions (72% of respondents). However, there has been a noticeable trend toward establishing new telestroke networks in middle-income regions, where nearly 90% of all networks set up in the past years are located. Network sizes ranged from far-reaching systems encompassing 60 hospitals and managing over 1200 consultations per year to smaller networks with only a few affiliated hospitals and around 100 consultations per year.

While teleconsultations’ primary purpose across networks was to guide thrombolysis and, to a lesser extent, thrombectomy, the technological implementation varied. Image transfer was a key component in most networks, but it is noteworthy that approximately 20% of networks conducted consultations without video examination.

There was a trend for network size and complexity to correlate with the economic status of the host country. Networks in HICs generally included more hospitals and consequently reported higher consultation volumes. Large-sized networks, of which 76% are in HICs, more often perform consultations with video examinations, image transfer, and written documentation than small-sized networks. In addition, these networks are more likely to have quality monitoring, standard operating procedures (SOPs), and research activities.

However, the survey revealed concerning gaps in quality assurance measures. Only 57% of networks could report their DNT, 57% measured in-hospital mortality, and only 41% monitor their recanalization rate—critical treatment effectiveness indicators. These findings highlight the need for more robust and consistent quality monitoring practices across telestroke networks to ensure optimal patient outcomes and facilitate continuous improvement in care delivery. Interestingly, although telestroke networks face several operational challenges, mainly of a technical nature, they are generally perceived as effective tools for improving stroke care delivery by increasing access to expert advice and improving the quality of care.

### Future implications

The considerable variability in telestroke models and the inconsistent reporting of performance indicators pose significant challenges in identifying the best practice in the area. To ensure sufficient quality standards are achieved, it is crucial to assess how many networks follow established telestroke recommendations, such as those of the European Stroke Organisation.^10^ Furthermore, a more comprehensive understanding of the barriers to telestroke implementation is essential. These barriers will likely vary in geographical and economic contexts and elucidating them is key to developing targeted strategies to expand telestroke coverage.

In recent years, there has been a notable shift in locations of new telestroke networks, with an increasing number being established in non-HICs. This trend highlights the need for globally applicable recommendations that consider the diverse realities of different regions, including different network sizes and levels of complexity. Such guidelines should be adaptable to different resource settings while maintaining core quality standards.

To address these challenges, we propose the development of a standardized operational framework for telestroke models, categorized as “basic,” “essential,” and “advanced,” considering the different resource availabilities. This tiered approach, aligned with the World Stroke Organization (WSO) roadmap,^21^ would provide a universally acceptable and structured pathway for telestroke development and implementation in different settings. By establishing these standards, we can promote more consistent and effective telestroke care worldwide, ensuring that networks at all levels of development have clear guidelines for operation and improvement. This approach would improve the quality of care and provide a roadmap for networks to develop and adapt to their specific contextual challenges and resources.

### Strengths and limitations

This study provides a comprehensive global mapping of telestroke coverage and offers unprecedented, detailed insights into networks across different geographical and economic regions. Our methodology employed a robust four-tiered strategy for network identification. With 254 identified networks, our identification process yielded more comprehensive results than previous literature reviews and surveys,^7–9^ observing similar distribution patterns. The survey had a response rate of 34%, which can be considered acceptable given the involvement of dedicated network leaders. The demographics of the respondents closely reflected the global distribution of telestroke networks across geographical regions. Most of our findings (89% of all responses) were derived from models implemented in HICs, Central Europe, and Latin America, reflecting the limited availability of telestroke care in other regions. Our analysis integrated both economic and health-related factors by utilizing two complementary classification systems: the World Bank’s income-based country categories and the GBD study’s epidemiologically focused super-regions. This dual approach provides a more comprehensive framework for examining global health patterns and disparities.

However, several limitations warrant consideration:

Definition of telestroke: First, the lack of a standardized definition of telestroke care, particularly regarding formal versus informal networks, may have led to the identification of predominantly structured networks. This bias could skew representation towards higher-income countries with more established telestroke initiatives, possibly overlooking less formal arrangements. While a 34% survey response rate is generally considered adequate, the global nature and high service variability identified may limit the generalizability of our findings.

We cannot guarantee the absolute credibility of self-reported data, although the fact that 60% of respondents were network coordinators with more than 5 years of experience enhances reliability. In addition, we found a high level of agreement between the survey responses and the published literature.^7,22,23^

We did not collect comprehensive baseline patient data or comprehensive network performance metrics beyond recanalization rates and DNTs. This limitation and incomplete reporting from some networks make comparative analysis and the definition of best practices difficult.

Finally, our study did not assess catchment areas, population densities, or distances between hospitals. These factors could provide additional valuable context for recommending appropriate telestroke models for different settings.

Despite these limitations, this study significantly advances our understanding of global telestroke coverage and operational characteristics. It provides a foundation for future research and policy development to optimize telestroke care delivery worldwide.

## Conclusion

This global survey of telestroke networks reveals significant disparities in coverage and implementation, with a clear bias toward HICs. However, the emerging trend of new networks in UMIC and LMICs signals a critical juncture in global telestroke care. More work is required in LMICs to implement telestroke services and support access to stroke reperfusion therapies. The striking inconsistency in quality monitoring practices across networks highlights the pressing need for standardized and resource-appropriate quality assurance measures adaptable to various settings.

Our findings underscore the urgent need for a paradigm shift in telestroke recommendations and the proposal of a specific framework for telestroke models aligned with the World Stroke Organization roadmap. This approach would provide adaptable, context-specific guidelines while maintaining core quality standards.

## Supplemental Material

sj-pdf-2-wso-10.1177_17474930241298450 – Supplemental material for Telemedicine networks for acute stroke: An analysis of global coverage, gaps, and opportunities Supplemental material, sj-pdf-2-wso-10.1177_17474930241298450 for Telemedicine networks for acute stroke: An analysis of global coverage, gaps, and opportunities by Christine Tunkl, Ayush Agarwal, Emily Ramage, Faddi Saleh Velez, Tamer Roushdy, Teresa Ullberg, Linxin Li, Leonardo A Carbonera, Abdul Hanif Khan Yusof Khan, Bogdan Ciopleias, Zhe Kang Law, Aristeidis H Katsanos, Mirjam R Heldner, Maria Khan, Sarah Matuja, Matias J Alet, Javier Lagos-Servellón, Jatinder S Minhas, Susanna M Zuurbier, Maria Giulia Mosconi, Radhika Lotlikar, Ahmed Elkady, Stefan T Gerner, Shirsho Shreyan, Alexandra Krauss, Christoph Gumbinger, Padma Srivastava, Pawel Kiper, Robin Ohannessian, Anne Berberich, Gisele Sampaio Silva and Anna Ranta in International Journal of Stroke

sj-pdf-3-wso-10.1177_17474930241298450 – Supplemental material for Telemedicine networks for acute stroke: An analysis of global coverage, gaps, and opportunities Supplemental material, sj-pdf-3-wso-10.1177_17474930241298450 for Telemedicine networks for acute stroke: An analysis of global coverage, gaps, and opportunities by Christine Tunkl, Ayush Agarwal, Emily Ramage, Faddi Saleh Velez, Tamer Roushdy, Teresa Ullberg, Linxin Li, Leonardo A Carbonera, Abdul Hanif Khan Yusof Khan, Bogdan Ciopleias, Zhe Kang Law, Aristeidis H Katsanos, Mirjam R Heldner, Maria Khan, Sarah Matuja, Matias J Alet, Javier Lagos-Servellón, Jatinder S Minhas, Susanna M Zuurbier, Maria Giulia Mosconi, Radhika Lotlikar, Ahmed Elkady, Stefan T Gerner, Shirsho Shreyan, Alexandra Krauss, Christoph Gumbinger, Padma Srivastava, Pawel Kiper, Robin Ohannessian, Anne Berberich, Gisele Sampaio Silva and Anna Ranta in International Journal of Stroke

sj-xlsx-1-wso-10.1177_17474930241298450 – Supplemental material for Telemedicine networks for acute stroke: An analysis of global coverage, gaps, and opportunities Supplemental material, sj-xlsx-1-wso-10.1177_17474930241298450 for Telemedicine networks for acute stroke: An analysis of global coverage, gaps, and opportunities by Christine Tunkl, Ayush Agarwal, Emily Ramage, Faddi Saleh Velez, Tamer Roushdy, Teresa Ullberg, Linxin Li, Leonardo A Carbonera, Abdul Hanif Khan Yusof Khan, Bogdan Ciopleias, Zhe Kang Law, Aristeidis H Katsanos, Mirjam R Heldner, Maria Khan, Sarah Matuja, Matias J Alet, Javier Lagos-Servellón, Jatinder S Minhas, Susanna M Zuurbier, Maria Giulia Mosconi, Radhika Lotlikar, Ahmed Elkady, Stefan T Gerner, Shirsho Shreyan, Alexandra Krauss, Christoph Gumbinger, Padma Srivastava, Pawel Kiper, Robin Ohannessian, Anne Berberich, Gisele Sampaio Silva and Anna Ranta in International Journal of Stroke
