# Supplementary material for: Telemedicine networks for acute stroke: An analysis of global coverage, gaps, and opportunities
Source: Int J Stroke. 2024 Nov 15;20(3):297–309. doi: 10.1177/17474930241298450 (PMC11874588; doi:10.1177/17474930241298450)
Supplement: sj-pdf-2-wso-10.1177_17474930241298450 – Supplemental material for Telemedicine networks for acute stroke: An analysis of global coverage, gaps, and opportunities [file sj-pdf-2-wso-10.1177_17474930241298450.pdf]

World Stroke Organization

WSO FSLP TeleStroke Survey

Join us in taking part in a survey led by the World Stroke Organization's Future Leaders.

Your valuable input will contribute to promoting the successful implementation of telestroke systems in remote or underserved areas.

If telestroke care is not yet available in your region, we also appreciate your feedback to Dr Christine Tunkl (christine.tunkl@med.uni-heidelberg.de). For any inquiries or concerns, please feel free to contact Dr. Christine Tunkl.

Thank you for your participation!

World Stroke Organization Future Leaders - Cohort II

We examine telestroke networks designed for use among ***healthcare professionals between hospitals*** *operating within the* ***emergency and acute inpatient hospital setting*** *(< 3 days after onset) and are* ***based on any formal processes*** *recognized by the hospitals.*
We exclude telerehabilitation networks and direct telemedicine consultations between doctors and patients, private counseling / guidance / mentorship / advice on an irregular or friendship basis or communication between resident and consultant within one hospital

Email:

1. In which country is your hospital?
* must provide value

2. What is your role in the network?
- Network coordinator
- Provider of telestroke care
- Caller / recipient of telestroke care
- Other

reset

3. How many years have you been involved in this telestroke network?
- < 1 year
- 1-5 years
- >5 year

reset

4. How many years has the network been in existence?

a. _____
b. In process of setting up

5. What kind of hospital support is provided by your telestroke model?
- 'hub-and-spoke' model
- Hubless network / horizontal
- Other - please specify:

reset

Hubs and spoke: stroke specialists at a central hub provide advice to regional spokes either prior to local treatment or transfer to the hub. Hubless' model: teleconsultations are performed by stroke specialists from the centres within the network

6. How many hospitals are connected in the network?

a. ___________
b. not sure

Expand

7. What is the location of most connected sites?
- Rural
- Urban
- Semi-urban
- Both
- I don't know

reset

8. Which of the following describes the setting of the originating hospital?
- Academic comprehensive stroke center
- Non-academic comprehensive stroke center
- Secondary care center
- No originating hospital exists
- For-profit company provides consultation
- Other - please specify:

reset

Originating hospital is the hospital providing teleconsultation Secondary care is specialist care provided on an ambulatory or inpatient basis, usually following a referral from primary care

9. Which of the following describes the setting of the remote / spoke hospital?
- Academic comprehensive stroke center
- Non-academic comprehensive stroke center
- Secondary care center
- Primary care center
- Other- please specify:

Primary care refers to the work of health professionals who act as a first point of consultation for all patients, e.g. family doctors, general practitioners. Secondary care is specialist care provided on an ambulatory or inpatient basis, usually following a referral from primary care.e.g. community hospitals without highly specialized services.

10. What is the purpose of tele-stroke?

Please tick all that apply
- Prehospital care
- Emergency consultation for(hyper-) acute stroke
- Hyperacute stroke management - thrombolysis
- Hyperacute stroke management - Endovascular thrombectomy
- Intracerebral hemorrhage - consultation
- Stroke unit supervision
- Rehabilitation
- Others: please specify

11. Which services are provided by your tele-stroke model?

Please tick all that apply
- Support in thrombolysis and thrombectomy decision-making
- Transfer for thrombolysis
- Transfer for thrombectomy
- Transfer for neurosurgical interventions
- Transfer for stroke unit care or intensive care
- Discussion of difficult cases
- Other:

12. What are the operating times of telestroke care provision?
- 24/7
- Out of core - working hours (e.g. nights, weekends)
- Special case advice only
- Other:

reset

13. How many telestroke consultation are provided on average by month (in the last year)?
- < 10
- 10 - 50
- 50 - 100
- > 100
- I don't know

reset

14. What is the average time needed for one complete consultation?
- < 5 minutes
- 5-20 minutes
- 20-45 minutes
- >45 minutes

reset

15. Do you have a standardized protocol (SOP)?
- Yes
- No
- I don't know

reset

16. What are the components of a teleconsultation?

Please tick all that apply
- Real-time video examination
- Voice call (physician - physician)
- Image transfer (CT / MRI)
- Written documentation (referral letter)
- Text messaging or whatsapp
- Other: please specify

17. Does your consultation include real-time video examination?
- Yes
- No

reset

18. Does your consultation include image sharing (CT/MRI)?
- Yes
- No

reset

19. Does your consultation include written documentation/reporting?
- Yes
- No

reset

20. What follow-up is provided after telestroke care by the telestroke provider/the originating hub?
- Follow up with the calling physician
- Follow up with the patient
- Only if required by the calling center
- None
- Other - please specify:

reset

21. What were the (estimated) costs for setting up the system (App, devices)?
a. ____
b. I don't know

Expand

22. What are the annual maintenance costs
- for the technical devices / App _______________
- for human resources ___________________
- I don't know

Expand

23. How many doctors in your institution provide telestroke care?
a. _________
b. I don't know

Expand

24. Do these physicians fill full-time positions for telestroke care only or do they offer teleconsults in addition to their regular jobs?
- Full-Time
- Part-Time/ Add-on
- I don't know
- Other:

reset

25. What is the qualification of the tele stroke consultant?

Please tick all that apply
- Resident Neurology
- Resident Internal Medicine
- Consultant Neurology
- Consultant Internal Medicine
- Other:

26. What is the average number of years of clinical experience of the consultant?
- < 1 year
- 1-5 years
- > 5 years

reset

27. What is the qualification of the person seeking tele-consultation?

Please tick all that apply
- Resident
- Qualified physician (e.g. internal medicine)
- Neurologist
- Nurses
- House officer
- Paramedical staff
- Emergency Physician
- General Practioner
- Patients

28. Is the thrombolysis (tPA) regional/ territorial rate being monitored?
- Yes
- No
- I don't know

reset

29. Is the quality and outcome of the telestroke program monitored?
- Yes
- No
- I don't know

reset

30. Does your network conduct any research within your network based on telestroke?
- Yes
- No
- I don't know

reset

31. How many patients get thrombolysis via teleconsultation per month on average (in the last year)?
a. _____________ (estimated / validated)
b. I don't know

Expand

32. How many patients are transferred to your center for EVT per month on average (in the last year)?
a. _____________ (estimated / validated)
b. I don't know

Expand

33. What is the average Door-to-Needle-Time via teleconsultation (in the last year)?
a. _____________ (estimated / validated)
b. I don't know

Expand

34. What is the financial model of the clinical telestroke collaboration?
- No financial transactions between the centers
- Funding received by the center where the patient is located and expert service paid to a public hospital
- Funding received by the center where the patient is located and expert service paid to a private hospital
- Funding received by the center where the patient is located and expert service paid to a private outsourced telestroke company or entity
- Funding received by the expert center and funds transferred to the center where the patient is located
- Other, please specify

reset

35. What is the funding model of the clinical telestroke activities?
- No additional funding for telestroke compared to standard stroke management funding
- Additional funding provided as a global budget independently of the volume of patients managed by telestroke
- Additional funding provided depending of the volume of patients managed by telestroke
- Out-of-pocket payment requested to the patient for management through telestroke
- Other: please specify

reset

36. Which barriers do you perceive in your telestroke network?

Please select all that apply
- Legal issues
- Issues with funding / financing
- Technical issues (app/ devices) with communication
- Technical issues with image transfer
- Internet connection
- Accountability in decision making
- Time exposure
- Too much documentation
- No clear procedure/ protocol
- Cooperation with caller
- Other - please specify:

37. Which strengths do you perceive in your telestroke network?
- Little time expenditure
- Improved quality of care in remote hospitals
- Less documentation
- Shorten referral time
- Able to get expert consultation
- Other - please specify:

38. What is your overall level of satisfaction with the tele-network model?
- Very satisfied
- Satisfied
- Neutral
- Dissatisfied
- Very dissatisfied

reset

39. Do you consider the quality of care provided by telecare in network is better, worse or equal to an onsite consultation?
- Much better
- Minimally better
- Equal
- Minimally worse
- Much worse

reset

Please share your email-address and the name of the network/ city/ region on a voluntary basis. This will help us to verify information in case of multiple, divergent responses from a network.

Email-Address:

Name of city/ network:

Expand

Do you agree that your network is listed in the WSO registry and in a publication?
- Yes
- No

reset

Do you agree to be contacted for future research questions?
- Yes
- No

reset

In case there are multiple networks in your country, please share their names and contact of their coordinator:

Submit

Save & Return Later

Powered by REDCap
