# Supplementary material for: Telemedicine networks for acute stroke: An analysis of global coverage, gaps, and opportunities
Source: Int J Stroke. 2024 Nov 15;20(3):297–309. doi: 10.1177/17474930241298450 (PMC11874588; doi:10.1177/17474930241298450)
Supplement: sj-pdf-3-wso-10.1177_17474930241298450 – Supplemental material for Telemedicine networks for acute stroke: An analysis of global coverage, gaps, and opportunities [file sj-pdf-3-wso-10.1177_17474930241298450.pdf]

Country	Region/ Network
Albania	University Hospital Mother Teresa
Argentina	Mendoza, Argentina
	San Miguel del Monte, Provincia de Buenos Aires
	Clínica Sagrada Familia with different provinces of Argentina
	Córdoba, Argentina
Austria	Salzburger Landeskliniken
Australia	South Australia and Northern Territory
	Victoria
	New South Wales
	Tasmania
	Queensland
	Western Australia
Belgium	Belgian Stroke Council, President of the Scientific Board
Brazil	Rede DASA
	Brasília / Rede D'Or
	Hospital M.Boi Mirim São Paulo
	Albert Einstein
Canada	Centre Hospitalier de l'Université de Montreal
	Ontario Telstroke System
	Winnipeg, Manitoba
	Vancouver, Western Canada
	Province of Alberta telestroke network
	University of New Foundland
	CHU de Québec - Université Laval network, Hôpital Enfant-Jésus, Québec city
	CHUM Montreal Dre Céline Odier and Hôpital de Chicoutimi
Chile	Chile (Santiago de Chile)
China	WenZhou Medical University
	Capital Medical University
	Sichuan Telestroke and Telethrombolysis Network, Western China
	Chengdu Medical College
Colombia	Teleictus Boyacá (tunja, Duitama, Sogamoso y Soata)
	Hospital Padrino ACV, Cali
Costa Rica	Costa Rica/San Jose
Croatia	University Hospital Center Zagreb
	University Hospital Center Sestre Milosrdnice (Zagreb
	University Hospital Center Rijeka
	University Hospital Center Split
	University Hospital Center Osijek

Denmark	Copenhagen+Bornholm and Faroe Islands
Dominican Republic	Northern Region
Ecuador	Ecuador
Egypt	Ain Shams University Telemedicine Telestroke network
Estonia	Hiiumaa
	Saaremaa
Ethiopia	
Finland	Finnish national Telestroke Service (HUH Neurology, Helsinki)
France	Bordeaux
	Comte
	Telestroke Franche Comté (University Hospital Jean Minjoz, Besançon)
	Telestroke Bourgogne (University Hospital, Dijon)
	Tele-Stroke Alsace (University Hospital, Strasbourg)
	TELENA : Tele-Stroke Nouvelle Aquitaine
	TELEMAAC (University Hospital, Clermont Ferrand)
	Tele-stroke basse normandie (University Hospital, Caen)
	Tele-stroke PACA (University Hospital, Nice)
	Telestroke Nord Pas de Calais (University Hospital, Lille)
	Telestroke Hainaut-Artois (General Hospital, Valenciennes/Lens)
	Telestroke Midi-Pyrénées (University Hospital, Toulouse)
Germany	Berlin / ANNOTeM
	Heidelberg / FAST
	Freiburg / FRITS
	Netzwerk Heide/Husum/Brunsbüttel
	Kassel / NeuroNetz Mitte
	Netzwerk Klinikum Region Hannover
	Hannover / MHH TNN
	München / NEVAS
	Netzwerk Klinikum Osnabrück
	Jena/ SATELIT
	Dresden / SOS-TeleNET
	Bayreuth, Nürnberg / STENO
	Karlsruhe / Stroke ARTEV
	TEMES-RLP
	München / TEMPiS
	Augsburg / TESAURUS
	Sachsen / TESSA
	Braunschweig / TN BC

	Würzburg / TRANSIT
Ghana	
Honduras	Honduras/Noroccidental
Hong Kong	
Hungary	National network
Iran	
Iraq	
Ireland	Mater Misericordiae Hospital, Dublin
	Tallaght-Naas telestroke
Italy	Telestroke USL Umbria 2 (Foligno Hospital, Foligno)
	Telestroke Lazio
	Reggio Emilia (Emilia Romagna)
	Toscana-Isola d'Elba
	Salerno
	Telestroke Treviso Conegliano (Hospital Conegliano Treviso ULSS 2)
India	Swasthaingit telestroke network
	Jammu and Kashmir State (Time to Act)
	Apollo Hospital Madurai and Karaikudi
	SMART India Project
	Chh. Sambhajinagar, Maharashtra
	AIIMS New Delhi
Japan	Tokyo
	Hokkaido University
Kazakhstan	National coordination center for emergency medicine in Astana
Kenya	Chak Stroke Network, Nairobi
Kyrgyztan	National Hospital
Laos	International with Thailand
Latvia	Riga / Latvia
Lithuania	Vilnius University Hospital Santaros Klinikos
	Republican Vilnius University Hospital
Malaysia	Sarawak
	Sabah
	Penang
Mexico	
	Punta de Mita Nayarir, México with UCSD USA
	Monterrey, Mexico
Mongolia	Ulaanbaatar of Mongolia

Morocco	Center Fez / Morocco
New Zealand	Central region
	Midlands
	South Islands
	North Islands
Netherlands	The Netherlands
Nigeria	
Norway	Vestreviken
	Norsk Luftambulanse
Paraguay	Hospital Central del IPS - Paraguay
	Hospital Prevision Social
Peru	Clínica SANNA San Borja - Red de clínicas SANNA - Perú
Portugal	Via Verde do AVC da região centro de Portugal
Russia	Sverdlovsk Telestroke Network (Sverdlovsk Regional Clinical Hospital #1, Ekaterinburg)
	Khakassian Telestroke Network (Republican Clinical Hospital named after G.Y. Remishevskaya, Abakan)
	Krasnodar Telestroke Network (Research Institute and Krai Clinical Hospital #1 named after S.V. Ochapovsky, Krasnodar)
	Krasnoyarsk Telestroke Network (Krasnoyarsk Interdistrict Clinical Hospital #20 named after I.S. Berzon, Krasnoyarsk)
	Novosibirsk Telestroke Network (Central Clinical Hospital No. 1, Novosibirsk, Novosibirsk)
	Primorsky Telestroke Network (Primorsky Krai Clinical Hospital #1, Vladivostok)
	Pskov Telestroke Network (Pskov Regional Clinical Hospital, Pskov)
	Yakutian Telestroke Network (Yakutsk Republican Clinical Hospital, Yakutsk)
Saudi Arabia	Seha Virtual Hospital network -MOH-Riyadh-
	Riadh Telestroke
Serbia	Telestroke Vojvodina (Clinic of Neurology, Clinical Centre of Vojvodina, Novi Sad)
Singapore	
Slovakia	
Slovenia	TeleKap network (University Medical Centre Ljubljana, Neurlogy Clinic, Ljubljana)
South Africa	
South Korea	
Spain	Complexo hospitalario universitario a coruna
	Hospital clinico universitario lozano blesa
	Hospital clinico universitario virgen de la arrixaca
	Hospital de leon
	Hospital Ruber Juan Bravo
	Hospital Universitari Germans Trias i pujol de Badalona
	Hospital Universitario Central de Asturias

	Telestroke Madrid (University Hospital, La Paz, Madrid)
	Hospital Universitario Fundacion Jimenez Diaz
	Hospital Universitario Miguel Servet
	Hospital Universitario y Politecnico La Fe
	Hospital Caceres
	Centro Andaluz de Teleictus, CATI
Sweden	South
	East
	Stockholm
	Gothenburg
	Örebro
	Uppsala
Switzerland	University hospital Bern
	University hospital Geneva
	University hospital Lausanne
	University hospital Basel
	Cantonal hospital Aarau
	University hospital Lucerne
	University hospital Zurich
	Hirslanden Zurich
	Cantonal hospital St. Gallen
	Cantonal hospital Lugano
	Hospital Nyon
	Cantonal hospital Sion
	Cantonal hospital Freiburg
	Cantonal hospital Neuenburg
	Hospital Biel
	Cantonal hospital Solothorn
	Hospital Schlieren
	Hospital Baden
	Hospital Winterthur
	Hospital Triemli
	Cantonal hospital Frauenfeld
	Hospital Münsterlingen
	Hospital Grabs
	Cantonal hospital Chur
Thailand	Neurological Institute of Thailand
	King Chulalongkorn Memorial Hospital
	Siriraj hospital

	Ramathibodi hospital
	Trang hospital
Turkey	
United Arab Emirates	
United Kingdom	East of England Stroke Telemedicine Partnership
	Cumbria & Lancs
United States	Ohio State Telestroke
	Blue Sky Telehealth
	University of Michigan
	UTHealth Houston
	Cedars-Sinai
	Mayo Clinic
	University of Colorado
	Columbia University
	Universtiy of California, San Diego
	UWMedicine
	University of Minnesota
	Medical Universtiy of South Carolina (MUSC)
	Washington University
	VA Natioanl TS Program (Veterans)
	Integris
	InTouch
	Northwestern Memorial Hospital
	Partners Healthcare
	Providence Health
	SOS Telemed / Access TeleCare
	University of Pittsburgh Medical Center
	University of Utah
	Vanderbilt University Medical Center
	Wake Forest Baptist Health
	Massachusettes General Hospital
	Avera
	Kaiser Permanente
	TeleSpecialist
	AmWell
	University of Virginia / UVA Health
	Intermountain Health, Utah
	West Virginia University

	Stanford Medicine
	University of New Mexico
	Louisiana
	Arkansas AR-SAVE
	Beth Israel Deaconess Medical Center in Boston
	Thomas Jefferson University, Philadelphia, USA.
	University of Georgia
	University of Rochester
	University of Tennessee
	University of Chicago
	University of Illinois, Chicago
	Duke University
	Keck Medicine of USC
	UCLA
	University of Pennsylvania
	Ascencion St John Medical Center
	University of Miami
	University of Oklahoma
	Univeristy of Cincinnati
	Hawaii
	Penn State Health
	University of Louisville Health
	AHN Alleghey General Hospital
Vietnam	Stroke Center - The 108 Military Central Hospital - Ha Noi
Yemen	Borg Al-Atiba Hospital Aden
Zambia	

Countries without acute telestroke services

Afghanistan	Mali
Algeria	Mozambique
Armenia	Myanmar
Bahrain	Nepal
Bangladesh	Nicaragua
Bolivia	Niger
Botswana	North Macedonia
Bulgaria	Oman
Burundi	Pakistan
Cameroon	Panama
Cuba	Papua-Neuguinea
Cyprus	Philippines
Czech Republic	Qatar
DR Congo	Romania
El Salvador	Samoa
Eswatini	Senegal
Fidji	Solomon Islands
Georgia	Sri Lanka
Greece	Sudan
Guatemala	Suriname
Iceland	Syria
Indonesia	Tanzania
Israel	Tonga
Jordan	Tunisia
Kiribati	Uganda
Kuwait	Ukraine
Libya	Uruguay
Madagascar	Vanuatu
Malawi	Venezuela

Countries without information on availability of telestroke services

Angola	Mauritius
Antigua and Barbuda	Mayotte
Azerbaijan	Moldova
Barbados	Namibia
Belarus	North Korea
Belize	Poland
Benin	Réunion
Bhutan	Rwanda
Bosnia and Herzegovina	Saint Helena
Brunei	Saint Kitts and Nevis
Burkina Faso	Saint Lucia
Cabo Verde	Saint Vincent and the Grenadines
Cambodia	San Marino
Central African Republic	Sao Tome & Principe
Chad	Seychelles
Comoros	Sierra Leone
Congo	Somalia
Côte d'Ivoire	South Sudan
Djibouti	State of Palestine
Equatorial Guinea	Taiwan
Eritrea	Tajikistan
Faeroe Islands	The Bahamas
French Guiana	Timor-Leste
Gabon	Togo
Gambia	Trinidad and Tobago
Grenada	Turkmenistan
Guinea	Uzbekistan
Guinea-Bissau	Western Sahara
Guyana	Zimbabwe
Haiti	
Lebanon	
Lesotho	
Liberia	
Luxembourg	
Macao	
Maldives	
Malta	
Mauritania	
